# Range and Elevational Shifts of Mistletoes Under Future Climate Change Scenarios

**DOI:** 10.1002/ece3.72388

**Published:** 2025-10-28

**Authors:** Antonio Acini Vásquez‐Aguilar, Saddan Morales‐Saldaña, Samantha Maite de los Santos‐Gómez, Andrea I. Barraza‐Ochoa, Juan Francisco Ornelas

**Affiliations:** ^1^ Red de Biología Evolutiva Instituto de Ecología, A.C. (INECOL) Veracruz Mexico

**Keywords:** climatic niches, elevational shifts, hemiparasites, Mexico, range shifts, species distribution modeling

## Abstract

Climate change is reshaping species' geographic distributions, with range shifts to higher elevations and latitudes. Parasitic plants like keystone mistletoes are particularly vulnerable to climate change because of their obligate dependence on host plants. Here we investigated how climate change under both optimistic and pessimistic scenarios will alter the distribution of suitable habitat of 10 *Psittacanthus* mistletoe species in Mesoamerica by 2050–2090. We assessed whether species with narrow habitat, geographic distribution, and host range face greater risks than generalist, widespread species. Suitable habitat for most temperate high‐elevation species shifted upward in elevation under most pessimistic climate scenarios, accompanied by significant range size reductions. These findings underscore the importance of evaluating climate change impacts on mistletoe distributions across diverse environments and biogeographical regions, as well as their ecological interactions with host plants and mutualists (pollinators and seed dispersers) to inform effective conservation strategies.

## Graphical abstract

Mistletoes are particularly vulnerable to climate change because of their obligate dependence on host plants. We investigated how climate change will alter the distribution of suitable habitat of Mesoamerican *Psittacanthus* mistletoes. Species differences were found in distribution with northward and upward shifts accompanied by significant range size reductions.

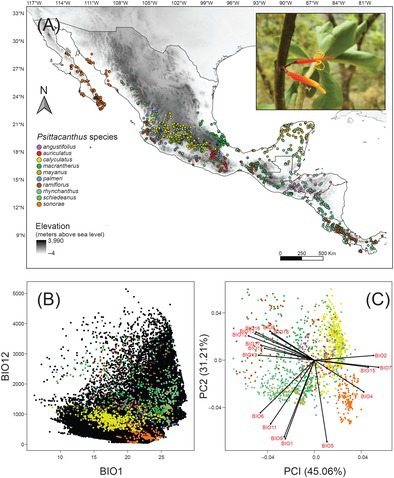

## 1 Introduction

Global climate change results from the emission of carbon dioxide and other greenhouse gases, deforestation, and other factors derived from shifting land use by human populations (Thomas et al. 2004; Zeppetello et al. 2020). These factors impact ecosystems differently in regions worldwide, with direct effects on life cycles, distribution, and ecology of numerous species and ecosystems (Thuiller 2007), many being unable to adapt to changing environmental conditions and, therefore, facing extinction (Merilä and Hendry 2014; Urban 2015). Current climate change is already altering species' geographic distributions, driving shifts to higher elevations and latitudes (Lu et al. 2023; Ramírez‐Barahona et al. 2025; Rojas‐Soto et al. 2012; Vásquez‐Aguilar et al. 2021), especially range‐restricted species (Abrha et al. 2018; Dávila et al. 2013; Ornelas, Licona‐Vera, and Vásquez‐Aguilar 2018; Xu et al. 2019).

Life history and physiological traits make parasitic plants sensitive to sudden climate changes because of their obligate dependence on host plants for minerals and water, and mobility (Cai et al. 2022; Mkala et al. 2022; Obico et al. 2024; Renjana et al. 2022; Zamora and Mellado 2019). The Santalales order has the largest species number (2428) among the 12 independent lineages of parasitic angiosperms, encompassing the widest array of nutritional modes that include autotrophic non‐parasites, hemiparasites, and holoparasites (Nickrent 2020). The stem or aerial parasites in Santalales, known as mistletoes (over 1600 species distributed globally; Nickrent 2020), are not a monophyletic group, and thus the term “mistletoe” refers to a functional group (Nickrent 2020; Watson 2001). Mistletoes parasitize host plants through an organ generally known as haustorium, forming a vascular connection to extract water and nutrients (Cocoletzi et al. 2016; Teixeira‐Costa 2021). Given their well‐documented ecological roles in nutrient cycling, forest stand dynamics, and provisioning of food and nest substrate, mistletoes are keystone species in many ecosystems (Watson 2001; Mathiasen et al. 2008). In addition, the complex interdependence of the mistletoes with host plants, pollinators, and seed dispersers makes them valuable bioindicators of ecosystem integrity (Griebel et al. 2017; Fontúrbel et al. 2018, 2021; Sangüesa‐Barreda et al. 2018).

Mistletoes are positively or negatively affected by anthropogenic changes. Their geographic ranges are threatened by habitat loss and habitat degradation (Fadini et al. 2024; Lapola et al. 2023), as well as exotic tree introductions and invasive species (Candia et al. 2014; Cuadra‐Valdés et al. 2021; Fadini et al. 2024; Sweetapple et al. 2002). The land‐use changes combined with climate change alter mistletoe ecological conditions at the community level by altering nutrient cycling and their interactions with their hosts and mutualists, both changing mistletoes' spatial structure, interaction effectiveness, facilitation process, interaction disruption, and novel interactions with invasive species (Fontúrbel et al. 2021; Walas et al. 2022; Watson et al. 2022). In contrast, the spreading facility of some mistletoe species in urban and semi‐natural areas, combined with their recent expansion to unoccupied regions due to climate change (Tikkanen et al. 2021), suggests that some mistletoes are fast‐spreading and growing species. Thus, mistletoes constitute an ideal model to determine their response to extreme climatic events.

While warming temperatures facilitate mistletoe expansion to higher latitudes and elevations (Dobbertin et al. 2005; Sangüesa‐Barreda et al. 2018; Tikkanen et al. 2021; Varga et al. 2014; Zamora and Mellado 2019), establishment is limited in areas that become too warm (Mellado and Zamora 2014; Varga et al. 2014), where water stress might compromise mistletoe floral and fruit traits essential for mutualistic interactions (Kuppler and Kotowska 2021; Reid and Kalcsits 2020). This is relevant for mistletoes that are not adapted to xeric conditions, in which parasitized host plants exposed to water shortage and severely affected by drought events would increase their mortality probability compared to non‐parasitized plants (Crates et al. 2022; Davidson et al. 1989; Moore and Lefoe 2020). Thus, the combined results of these intersecting stressors affect interaction networks and, if they fail to adapt, rearranged networks may precipitate community‐wide extinction cascades (Fontúrbel 2020; Fontúrbel et al. 2018, 2021; Watson et al. 2022). Under future warming scenarios, species are expected to migrate towards cooler, higher latitudes and elevations and, if needed, host switching in mistletoes (Watson et al. 2022; Zamora and Mellado 2019). However, this is challenging for mistletoes, as they depend on other species, which may have different dispersal abilities to shift their distribution ranges (Rai et al. 2018; Sangüesa‐Barreda et al. 2018) and, thus, mistletoe persistence would be precluded by spatial mismatches with their hosts, pollinators, and seed dispersers (Fontúrbel 2020; Ornelas, Licona‐Vera, and Ortiz‐Rodriguez 2018).

Two previous studies have evaluated the influence of climate change in Europe on the distribution of two widespread mistletoe species (Walas et al. 2022; Baranowska et al. 2025). Walas et al. (2022) found that three *Viscum album* L. (Viscaceae) subspecies differ in their responses to temperature, a key determinant of their future potential ranges. While *V. album* subsp. *abietis* (a parasite of *Abies* spp.) is withdrawing from its current range and *V. album* subsp. *austriacum* (a parasite of *Pinus* spp.) is shifting its range slightly, the host generalist and most widespread subspecies *V. album* subsp. *album* is widening its range area non‐directionally (Walas et al. 2022). The results of Baranowska et al. (2025) predict significant range expansions into northern and eastern Europe for the widespread *Loranthus europaeus* Jacq. (Loranthaceae) under future climate change scenarios, with climatic variables (winter and spring frosts) and host plant distribution (*Quercus* spp.) having the highest impact on potential distribution models for the mistletoe *L. europaeus* in Europe (Baranowska et al. 2025). These findings suggest that, akin to *V. album* subsp. *austriacum* (Walas et al. 2022), *L. europaeus* could become a substantial factor negatively impacting forests in Central Europe.

Here, we assess the potential effects of future climate change on the distribution of 10 Mesoamerican *Psittacanthus* Mart. (Santalales: Loranthaceae) mistletoe species using species distribution and ecological niche modeling. We tested the hypothesis that the potential future distribution of range‐restricted *Psittacanthus* species would be more constrained because of their narrow environmental tolerances compared to widely distributed species. According to the mistletoe threat index (Fadini et al. 2024), we further hypothesize that endemic specialist mistletoes occurring only in one habitat type, host‐range restricted, have a restricted distribution, and that rely on one pollinator/seed disperser mutualist species, would be more threatened and will show a decline in their species range under future climate change conditions, than generalist mistletoe species in their mutualistic interactions, with a much wider host range, geographically widespread, and distributed across different habitat types (Fontúrbel 2020; Reid and Lange 1988). We aimed to (1) estimate the current potential distribution of 10 Mesoamerican *Psittacanthus* species throughout their geographic range, (2) predict the potential impact of climate change on its geographical and elevational distribution, and to assess (3) whether the density of climatic niche is related to the predicted future changes in the geographical distribution and (4) the degree of niche overlap between species in a phylogenetic context.

## 2 Materials and Methods

### 2.1 Study System

*Psittacanthus* (*c*. 110 currently recognized species; Dettke and Caires 2021) is distributed from northern Mexico to northern Argentina and Brazil. These photosynthetic hemiparasites with large and bulky haustoria (Cocoletzi et al. 2016) are distinguished by their long colorful flowers pollinated mainly by hummingbirds and few species by bees and bats (e.g., Castro et al. 2023; Diniz et al. 2022; Fadini et al. 2018; Guerra et al. 2014; Pérez‐Crespo, Lara, and Ornelas 2016; Ramírez and Ornelas 2010), and by their large, lipid‐rich, one‐seeded fruits consumed and dispersed by birds (López de Buen and Ornelas 1999, 2001; Lara et al. 2009; Ramírez and Ornelas 2012).

We investigated the geographical and altitudinal range of 10 Mesoamerican *Psittacanthus* species (see Table S1). Our analysis included both narrowly distributed and widespread species and encompassed key ecological traits such as habitat specialization and host range. The phylogenetic distribution of these characteristics is presented based on the working phylogeny of *Psittacanthus* (see further details in Ornelas et al. 2024).

### 2.2 Data Collection

**FIGURE 1:**
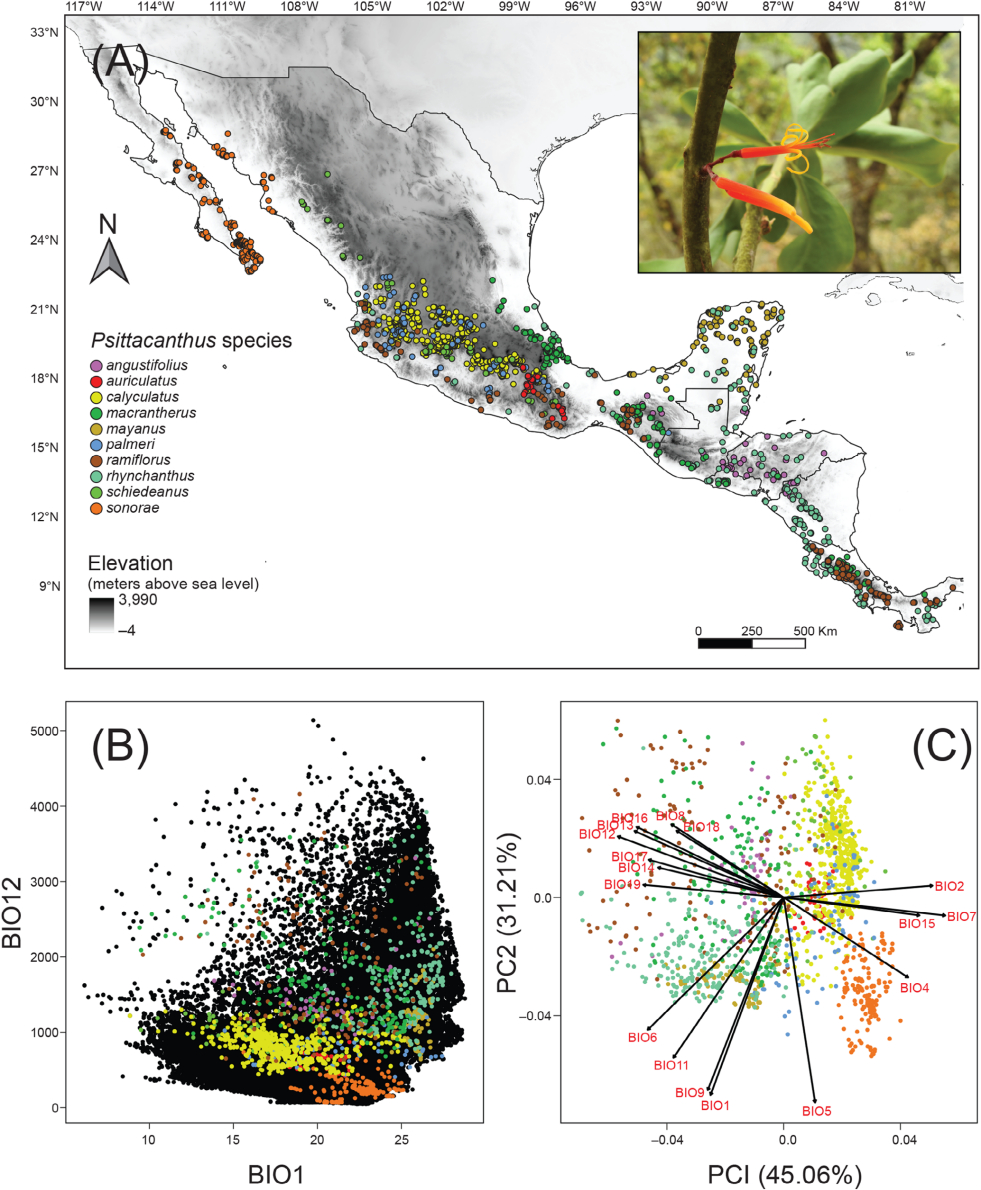
Environmental variation across the ranges of *Psittacanthus* mistletoes. (A) Relief map showing the location of the occurrence records (dots) used for ecological niche models and the distribution ranges of 10 *Psittacanthus* species. Colors of dots on the map are based on the principal components analysis (PCA) of the bioclimatic data shown in (C). *Psittacanthus ramiflorus* in the inset (photo by Ernesto A. López Huicochea). (B) Scatterplot using annual mean temperature (BIO1) and annual precipitation (BIO12) with the background environment of the historical area of accessibility (“M”) created for the 10 *Psittacanthus* species. Background points (black) represent 50,000 randomly sampled points across the distribution range of *Psittacanthus* focal species. Points from each species are represented by different colors as in (A). (C) Biplot of the PCA of bioclimatic data indicating the correlations among variables and the direction and magnitude of their contribution to the first two PCs of the PCA.

We downloaded all geographic occurrences for 10 species of *Psittacanthus* mistletoes (Figure 1A, Table S1) from the Global Biodiversity Information Facility (GBIF, www.gbif.org), removing records with geographic or taxonomic inconsistencies. GBIF raw occurrence records can be downloaded from: https://doi.org/10.15468/dl.d89trm (*Psittacanthus angustifolius*), https://doi.org/10.15468/dl.8tpta2 ( *P. auriculatus* ), https://doi.org/10.15468/dl.6m7453 (*P. calyculatus*), https://doi.org/10.15468/dl.xshcfg (*P. macrantherus*), https://doi.org/10.15468/dl.zuaara ( *P. mayanus* ), https://doi.org/10.15468/dl.nnytq5 (*P. ramiflorus*), https://doi.org/10.15468/dl.mjrnqw (*P. rhynchanthus*), 10.15468/dl.6f33zz (*P. schiedeanus*), https://doi.org/10.15468/dl.nnytq5 ( *P. palmeri* ), and https://doi.org/10.15468/dl.k8hx99 ( *P. sonorae* ). We supplemented the GBIF dataset with georeferenced records from field collection of several studies (Howell and Mathiasen 2004; Kuijt 2009; Licona‐Vera et al. 2018; Mathiasen et al. 2000; Mathiasen et al. 2007; Ornelas et al. 2016; Ornelas, Licona‐Vera, and Vásquez‐Aguilar 2018; Ornelas et al. 2019; Ortiz‐Rodriguez et al. 2020; Pérez‐Crespo et al. 2017).

Each species dataset was downloaded and cleaned up separately. Georeferenced data were checked for errors to ensure data quality, and geographical coordinates were revised for data consistency with the species' historical range. Each dataset was verified spatially to remove duplicate points using Wallace 2.2 (Kass et al. 2023), ArcMap 10.5 (ESRI 2016), and QGis 3.8.3 (QGIS Development Team 2019), excluding duplicate occurrence records or records near to each other (*c*. 2 km^2^) to reduce the effects of spatial autocorrelation (see Data Availability Statement for link to download occurrence data points and geographic information). After careful verification of every data location, we restricted the species datasets to 1851 unique presence records used to generate current and future species distribution models (SDMs), with a final number of occurrences per species ranging from 43 to 716 records (Figure 1A; see data for unique presence records in Table S1).

### 2.3 Environmental Data

We used 19 bioclimatic variables summarizing precipitation and temperature data (Table S2) as climate layers obtained from the WorldClim 2.1 database (Booth et al. 2014; Fick and Hijmans 2017) at 1 km^2^ spatial resolution for the average period 1970–2000. We explored three datasets based on different criteria for the selection of bioclimatic variables: (set 1) removal of one variable from each pair of variables for which Pearson product–moment correlations were high (*r* ≥ 0.8); (set 2) identification of variables that contribute most strongly to models using the jackknife in MaxEnt 3.4.1 (Phillips et al. 2006, 2017); and (set 3) inclusion of only variables with variance inflation factor (VIF) values less than 10 (following Brauner and Shacham 1998; Guisan et al. 2002). Note that the most relevant bioclimatic variables used for predicting the current distribution varied among species (Table S3).

For model building, the optimal parameters were selected using the kuenm package in R (Cobos et al. 2019). Model calibration entailed the evaluation of candidate models created with 11 distinct regularization multipliers (from 0.1 to 2.1, at intervals of 0.2), different combinations of four feature classes: linear, quadratic, product, and threshold (l, q, p, t, lq, lp, lt, qp, qt, pt, lqp, lqt, lpt, qpt, lqpt), and using the three sets of environmental variables (set 1, set 2, set 3) with a distinct number of variables each. Best parameter settings were selected considering the statistical significance of the partial Receiver Operating Characteristic (ROC), the predictive power (omission rates *E* = 5%), and the complexity level with the Akaike Information Criterion (AIC) corrected for small sample sizes (Anderson et al. 2003; Peterson et al. 2008; Warren et al. 2008).

### 2.4 Environmental Characterization of *Psittacanthus* Species

We compiled a dataset of 1851 occurrence records encompassing currently recognized ranges of 10 Mesoamerican *Psittacanthus* species (Figure 1A) and data for 19 bioclimatic variables from the WorldClim database 2.1 at ~1 km^2^ spatial resolution (Fick and Hijmans 2017; https://www.worldclim.org). To differentiate the environmental space among *Psittacanthus* species, we first characterized the background environment of the historical area of accessibility (area “M”; from the BAM diagram; Soberón and Peterson 2005). The characterization of the set of sites accessible to a species over which models are calibrated (Atauchi et al. 2020; Barve et al. 2011; Freeman et al. 2019; Soberón and Peterson 2005) was done by generating a total of 50,000 randomly sampled points within the area “M” created for the 10 *Psittacanthus* species. Calibration of SDMs for each species was done by geographical clipping based on the shapefile of the corresponding ecoregions from the World Wildlife Fund (Olson et al. 2001), representing potential boundaries on the landscape to dispersal (Barve et al. 2011; Machado‐Stredel et al. 2021). For each species, we selected those ecoregions where at least one point of occurrence was found. Following calibration, we masked all environmental variables to the extent of the “M” area in ArcMap 10.5 (ESRI 2016). For present‐day projections, we used the “M” area of each *Psittacanthus* species, whereas for future climate projections, we projected the models onto a broader M area, incorporating a 20 km buffer to account for potential dispersal scenarios. Then, we extracted values of each of the 19 bioclimatic variables for all occurrence records and background points using the “raster” R package (Hijmans et al. 2025; https://rspatial.org/raster). Subsequently, we visualized species in a scatterplot using annual mean temperature (BIO1) and annual precipitation (BIO12). Lastly, we used data on the 19 bioclimatic variables to carry out a principal component analysis (PCA) to identify the combination of bioclimatic variables that best separates *Psittacanthus* species.

### 2.5 Selection of Climate Models to Estimate Future Distribution

For future climate projections (years 2050, 2070, and 2090), we used the WorldClim 2.1 data from the Coupled Model Intercomparison Project 6 (CMIP6; Stoerk et al. 2018), at 1 km^2^ [*c*. 30 s of arc (arcmin)] spatial resolution (0.93 × 0.93 km = 0.86 km^2^ at the equator). We used predictions of the general circulation models under the shared socioeconomic pathways (SSPs) proposed by CMIP6 to predict future climate change (Popp et al. 2017).

We selected three different global climate models (GCMs) using GCM compareR (Fajardo et al. 2020) that represent specific future climate conditions (Zappa and Shepherd 2017): (i) high temperature and low precipitation compared to the ensemble projection (CanESM5), (ii) low temperature and high precipitation compared to the ensemble projection (MIROC6), and (iii) temperature and precipitation close to the average ensemble projection (IPSL‐CM6A‐LR). To predict the future spatial distribution of *Psittacanthus* species, we selected two Shared Socio‐economic Pathways scenarios (SSP1‐2.6 and SSP5‐8.5) representing the minimum and maximum greenhouse gas emission scenarios and climate change mitigation policies (Riahi et al. 2017).

### 2.6 Spatial Model Procedures and Validations

We generated the final SDMs with MaxEnt 3.4.1 (Phillips et al. 2006, 2017), using occurrence records and climatic variables with ten cross‐validation replicates and the results of model calibration obtained from the kuenm package in R (Cobos et al. 2019). Models were run with no extrapolation and no clamping to avoid artificial projections of extreme values of ecological variables (Elith et al. 2011; Owens et al. 2013), ensuring only one locality per grid cell. We randomly selected 75% of occurrence records for the training set and 25% for the test set (Gong et al. 2022). The algorithm performed 1000 iterations of these processes or continued until convergence (threshold: 0.00001; Phillips and Dudík 2008). We used the MaxEnt algorithm over other available frameworks for species distribution modeling because it is still among the top‐performing models and suitable for presence‐only data (Elith et al. 2006, 2011; Qiao et al. 2016; Valavi et al. 2022).

To produce the final present‐day ensemble maps, we calculated the median value across 10 algorithmic repetitions of each species. Subsequently, for future climate scenarios, we generated consensus projections for each combination of global climate model (CanESM5, MIROC6, and IPSL‐CM6A‐LR) and shared socioeconomic pathway (SSP). This was achieved by averaging the projections for each scenario using the Raster calculator tools in ArcMap. The distribution of species areas for current and future projections were calculated (in pixels) for the different climate change scenarios (SSPs) for the years 2050, 2070, and 2090, considering only the pixels with presence values (Vásquez‐Aguilar et al. 2021). We converted the differences in the mean number of cells of potential habitats to surface area (km^2^). To assess range shifts in response to climate change of each *Psittacanthus* species, we extracted elevation values from 10,000 random geographic points from each of the models (present, future with two SSPs). Then, ridgeline plots (formerly joyplots) showing elevation above sea level of 10,000 random points extracted from the distribution models were constructed using the ggplot2 package in R.

The R scripts used to perform the analyses, as well as the dataset of total occurrence data points and shape files, are available for download from https://doi.org/10.5281/zenodo.15307295.

### 2.7 Niche Quantification

To quantify climatic niche divergence among *Psittacanthus* species, we performed niche overlap analyses in R using the *ecospat* package (Di Cola et al. 2017). This analysis employs the PCAenv method (Broennimann et al. 2012), which projects species occurrence onto a two‐dimensional environmental space by the first two axes of Principal Components Analysis (PCA). The PCA was calibrated on the pooled environmental data across the study extent (the same used for SDM calibration; Table S3).

Niche overlap was quantified using Schoener's *D* (Schoener 1970) and Warren's *I* (Warren et al. 2008) metrics, both of which range from 0 (no overlap) to 1 (complete niche overlap). The density of occurrences within the environmental space for each species was visualized using the ecospat.plot.niche function (Di Cola et al. 2017) in R. Furthermore, we employed two statistical tests to interpret overlap metrics: an equivalency test to determine if the observed niches are statistically identical and a similarity test to assess whether the observed niche overlap is more similar or different than expected by chance given the available environmental background. Each randomization process was repeated 100 times, producing a null distribution of overlapping values against which the observed score was compared. A niche equivalency test was rejected if the observed Schoener's *D* value fell outside the 95% confidence interval of the stochastically simulated values (*p* < 0.05), indicating that the ecological niches of the two species are not equivalent. For the similarity test, we reject the null hypothesis that niche overlap is explained by regional habitat availability if the observed similarity between species falls outside the 95% confidence limits of the simulated null distribution.

To illustrate the contribution of each of the variables, we elaborated a heatmap showing the BIO variables that most contributed to each *Psittacanthus* model using the average values of 10 MaxEnt replicates. All statistical analyses were performed in R 4.4.1 (R Development Core Team 2024).

## 3 Results

### 3.1 Environmental Characterization

*Psittacanthus* species differed in their climatic affinities (Figure 1B). Species from xeric environments ( *P. auriculatus* , *P. sonorae* , *P. palmeri* ) occupied environmental spaces restricted to low annual precipitation but a wide range of annual mean temperatures. In contrast, *P*. *ramiflorus* and *P*. *rhynchanthus* occupied a wider environmental space in terms of annual mean temperature and precipitation (Figure 1B). PCA analysis identified two components that explain 76.27% of the total variance in the data (PC1 = 45.06%, PC2 = 31.21%), identifying three clusters with different environmental affinities (Figure 1C). Species *P. auriculatus* , *P. sonorae* , *P*. *calyculatus*, and *P. palmeri* were associated with mean diurnal range (BIO2), temperature seasonality (BIO4), temperature annual range (BIO7), and precipitation seasonality (BIO15) on the positive side of axis 1. In contrast, *P. angustifolius* , *P*. *ramiflorus*, and *P*. *schiedeanus* were associated with annual mean temperature (BIO1), min temperature of coldest month (BIO6), mean temperature of driest quarter (BIO9), and mean temperature of coldest quarter (BIO11). Conversely, *P. mayanus* , *P*. *macrantherus*, and *P*. *rhynchanthus* were associated with the mean temperature of wettest quarter (BIO8), annual precipitation (BIO12), precipitation of wettest month (BIO13), precipitation of driest month (BIO14), precipitation of wettest quarter (BIO16), precipitation of driest quarter (BIO17), precipitation of warmest quarter (BIO18), and precipitation of coldest quarter (BIO19) (Figure 1C).

### 3.2 Current Potential Distribution

The parameter optimization results for the 10 *Psittacantus* species generated 495 candidate models per species, each utilizing distinct combinations of climatic variables (Table S3). From this suite of models, we selected the single best‐performing model for each species based on statistical significance, the partial ROC, omission rate, and AICc criteria (Table S4). The optimal feature class combinations and regularization multiplier values were estimated independently for each *Psittacanthus* species (Table S4).

**FIGURE 2:**
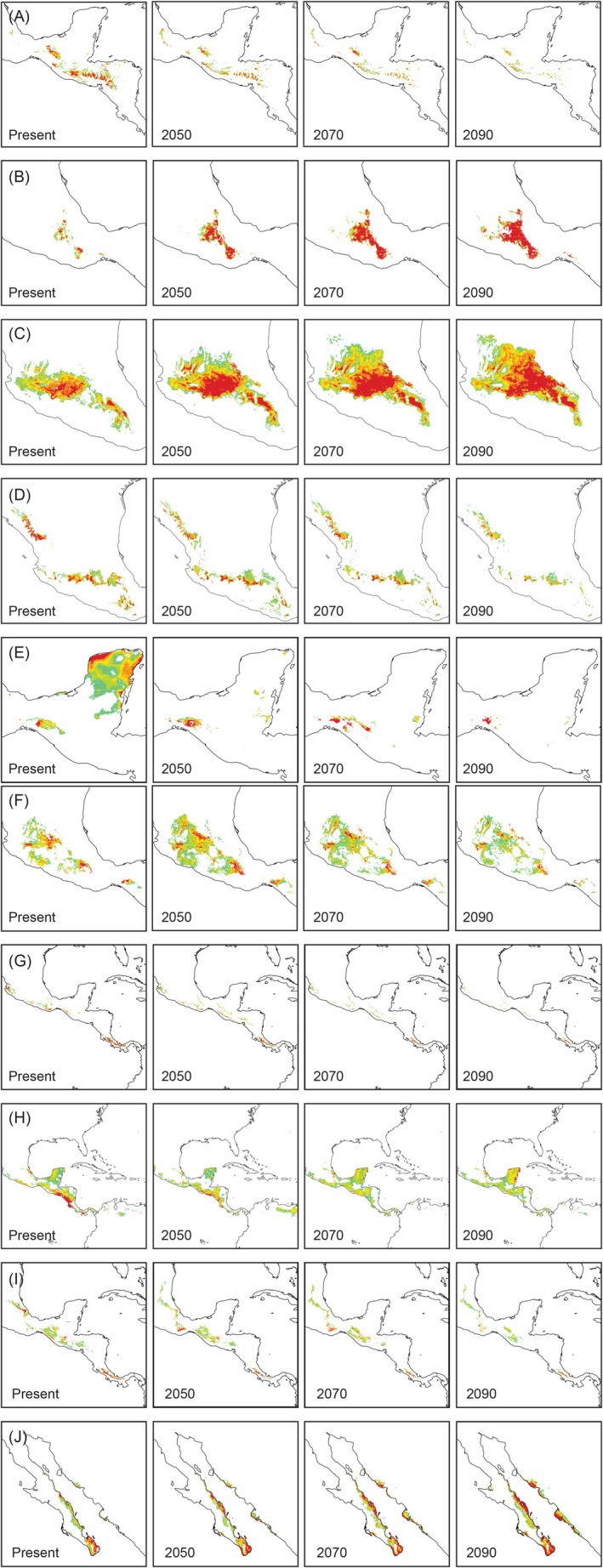
Predicted distribution of *Psittacanthus* mistletoe species under climate‐change scenarios, from pessimistic SSP 8.5 Shared Socioeconomic Pathways conditions for 2050, 2070, and 2090. (A) *Psittacanthus angustifolius*, (B) *P. auriculatus* , (C) *P*. *calyculatus*, (D) *P*. *macrantherus*, (E) *P. mayanus* . Predicted distribution of *Psittacanthus* mistletoe species under climate‐change scenarios, from pessimistic SSP 8.5 Shared Socioeconomic Pathways conditions for 2050, 2070, and 2090. (F) *Psittacanthus palmeri*, (G) *P*. *ramiflorus*, (H) *P*. *rhynchanthus*, (I) *P*. *schiedeanus*, (J) *P. sonorae* .

The predicted suitable habitats for all 10 *Psittacanthus* species were consistent with their known distributions, but were generally fragmented (Figure 2, Figure S1). The potential geographical range sizes varied substantially among species, ranging from 10,605 km^2^ ( *P. auriculatus* ) to 637,617 km^2^ (*P*. *rhynchanthus*) (Figure 2; Table 1). Out of the five species from xeric and lowland environments ( *P. auriculatus* , *P. mayanus* , *P. palmeri* , *P*. *rhynchanthus*, *P. sonorae* ), *P. auriculatus* had the smallest range size area (10,605 km^2^) (Figure 2B). The range areas of the species distributed in cloud forests, oak forests, pine‐oak forests, or pine‐fir forests at higher elevations ( *P. angustifolius* , *P. calyculatus*, *P. macrantherus*, *P. ramiflorus*, *P. schiedeanus*) were intermediate in size, ranging from 63,581 km^2^ ( *P. angustifolius* ) to 125,736 km^2^ (*P*. *macrantherus*) (Table 1). *Psittacanthus calyculatus*, *P. macrantherus*, *P. mayanus* , *P. palmeri* , *P. rhynchanthus*, and *P. schiedeanus* had the largest range size and widest distributions, whereas *P. angustifolius* , *P. auriculatus* , *P. ramiflorus*, and *P. sonorae* had the most restricted range size (Figure 2, Figure S1; Table 1 and Table S1).

**TABLE 1:** Predicted increase or decrease (%) in the extent of current suitable areas (km^2^) for 10 *Psittacanthus* species and under climate‐change scenarios, from optimistic (SSP 2.6) to extreme (SSP 8.5) Shared Socioeconomic Pathways conditions for the years 2050, 2070, and 2090.

Species	Models												
	Present	2050				2070				2090			
		2.6		8.5		2.6		8.5		2.6		8.5	
	Area	Area	%	Area	%	Area	%	Area	%	Area	%	Area	%
*P. angustifolius*	63,581	44,238	−30.4	37,726	−40.7	42,997	−32.4	26,235	−58.7	44,991	−29.2	15,512	−75.6
*P. auriculatus*	10,605	21,618	103.8	37,422	252.8	20,444	92.7	26,408	149.0	34,486	225.1	38,632	264.2
*P. calyculatus*	127,287	160,801	26.3	180,013	41.4	157,632	23.8	204,110	60.4	158,010	21.8	216,931	70.4
*P. macrantherus*	125,104	107,392	−14.1	97,711	−21.9	111,072	−11.2	76,166	−39.1	105,949	−15.3	46,673	−62.7
*P. mayanus*	119,540	27,080	−77.3	32,615	−72.7	61,990	−48.1	12,653	−89.4	12,081	−89.8	2772	−97.6
*P. palmeri*	105,736	197,456	86.7	189,732	79.4	190,827	80.4	191,889	81.4	153,755	45.4	125,101	18.3
*P. ramiflorus*	65,541	81,185	23.9	64,501	−1.6	80,925	23.5	34,365	−47.6	78,630	20.0	14,665	−77.6
*P. rhynchanthus*	637,617	654,054	2.6	609,017	−4.5	650,006	1.9	651,592	2.2	649,887	1.9	633,239	−6.9
*P. schiedeanus*	104,764	106,332	1.5	93,777	−10.5	100,902	−3.7	79,211	−24.4	107,966	3.1	52,889	−49.5
*P. sonorae*	27,640	32,093	35.0	34,189	43.8	28,690	20.6	31,826	33.8	38,156	60.5	47,821	101.1

### 3.3 Future Climate Change Projections

SDMs predicted that suitable habitat for the studied *Psittacanthus* species will decline or expand under both SSP 2.6 (optimistic) and SSP 8.5 (pessimistic) scenarios by 2050–2090, with the most pronounced shifts occurring by 2090 (Figure 2, Figure S1; Table 1). Under the most pessimistic SSP 8.5 conditions for 2090, species with xeric affinities exhibited potential range expansions, suggesting increased habitat suitability in arid environments (18.3% in *P. palmeri* to 264.2% in *P. auriculatus* ), while high‐elevation species face significant range contractions (−49.5% in *P*. *schiedeanus* to −77.6% in *P*. *ramiflorus*) (Figure 2, Figure S1; Table 1). Predictions for three species (*P. calyculatus*, *P. mayanus* , and *P. rhynchanthus*) showed opposite trends (Table 1), with an expansion of suitable areas for *P*. *calyculatus* (70.4%; Figure 2C), a reduction for *P. mayanus* (−98%; Figure 2E), and minimal changes for *P*. *rhynchanthus* (−6.9% to 2.6%; Figure 2H). Among the species experiencing a reduction in the distribution of suitable areas, losses ranged from 7% to 98% (Table 1), with *P. mayanus* being the most affected (Figure 2E; Table 1), followed by *P. ramiflorus* (78%) and *P. angustifolius* (76%). In contrast, species gaining distribution area showed increases ranging from 18% to over 200% of their current distribution (Table 1), with *P. auriculatus* exhibiting the greatest expansion, primarily across the southern portion of its current range and connecting its current fragmented distribution (Figure 2B; Table 1).

**FIGURE 3:**
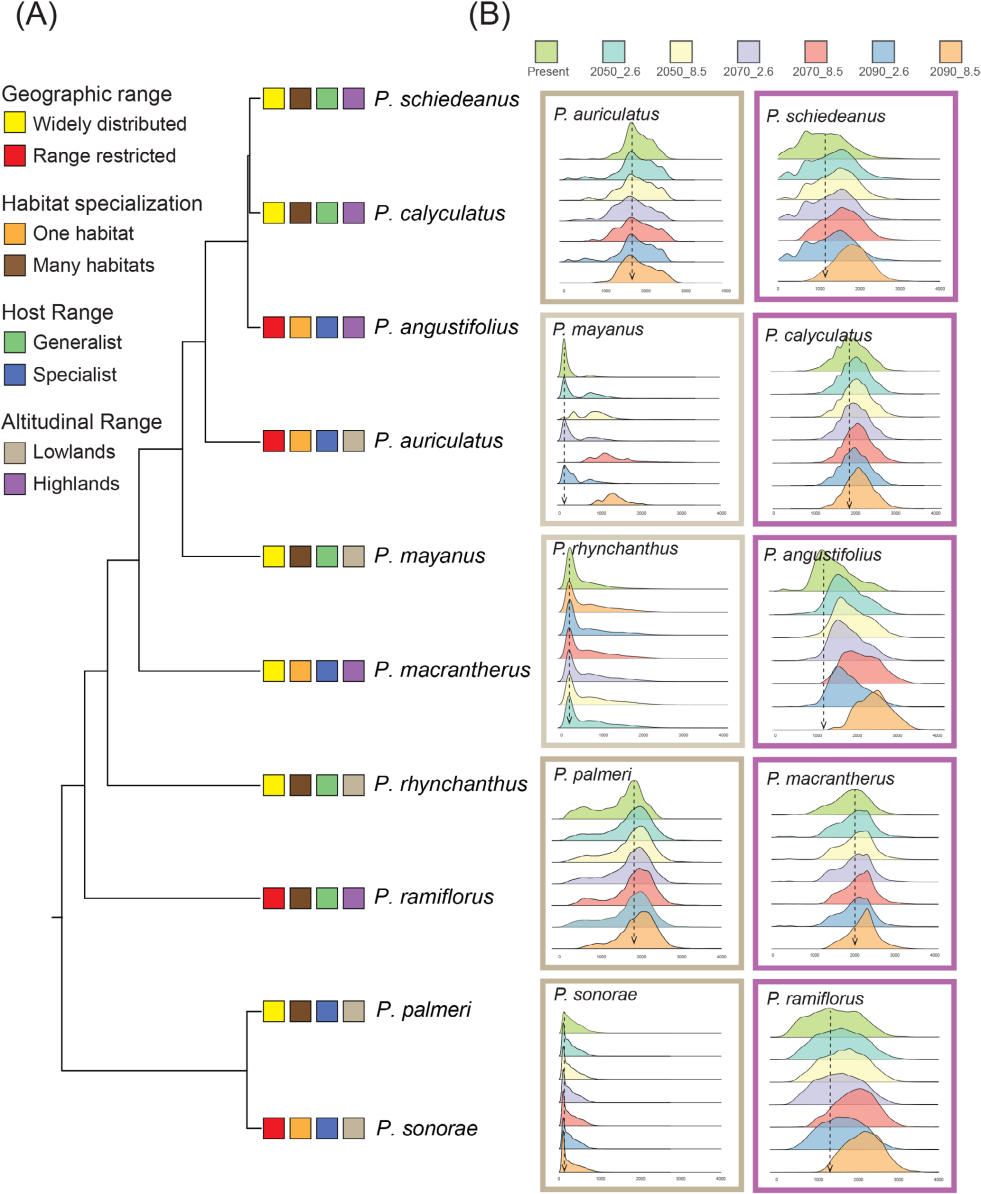
(A) Phylogenetic tree of *Psittacanthus* mistletoes along with (B) results of predicted elevational distribution of *Psittacanthus* mistletoe species under current and future climate‐change scenarios, from optimistic SSP 2.6 to pessimistic SSP 8.5 Shared Socioeconomic Pathways conditions for 2050, 2070, and 2090. Tree phylogeny (A) was derived from a working phylogeny led by J. F. Ornelas (Ornelas et al. 2024). *Psittacanthus* mistletoe species coded in the phylogeny by geographic range (widely distributed and range restricted), habitat specialization (one habitat and many habitats), host range (generalist and specialist), and by elevational range (lowlands and highlands). Results presented in the left panel (light brown) correspond to species distributed in xeric and tropical environments at lower elevations and the right panel (purple) to species distributed in more temperate forests at higher elevations. See species coding in Table 1.

Our analyses reveal consistent upslope range shifts for most species from present to 2090, with species at higher elevations exhibiting more pronounced elevational shifts compared to lowland, xeric, or tropical species (Figure 3; Table 2). Species from xeric environments, *P. auriculatus* (21 m upslope) and *P. sonorae* (48 m downslope), exhibited minimal elevational changes compared to species particularly distributed at higher elevations (Figure 3; Table 2); these species are distributed in mid‐elevation xeric environments and are restricted to a single habitat, and are host specialists. In contrast, species distributed at higher elevations exhibited the greatest upslope expansion on average, ranging from 191 m upslope in *P*. *calyculatus* to 982 m in *P. angustifolius* (Figure 3; Table 2).

**TABLE 2:** Predicted elevational shifts (m above sea level) of suitable areas (Avg = average, Med = median) for 10 *Psittacanthus* species under climate‐change scenarios, from optimistic (SSP 2.6) to extreme (SSP 8.5) Shared Socioeconomic Pathways conditions for the years 2050, 2070, and 2090.

Species	Models													
	Present		2050				2070				2090			
			2.6		8.5		2.6		8.5		2.6		8.5	
	Avg	Med	Avg	Med	Avg	Med	Avg	Med	Avg	Med	Avg	Med	Avg	Med
*P. angustifolius*	1306	1227	1626	1569	1732	1675	1642	1580	1981	1941	1642	1577	2288	2285
*P. auriculatus*	1759	1735	1723	1739	1728	1729	1605	1610	1731	1712	1681	1714	1780	1723
*P. calyculatus*	1897	1894	1995	1994	2028	2020	1985	1974	2055	2044	1983	1976	2088	2075
*P. macrantherus*	2087	2126	2188	2240	2248	2295	2183	2246	2335	2366	2208	2268	2451	2479
*P. mayanus*	110	30	395	160	695	729	316	124	1163	1066	298	170	1230	1207
*P. palmeri*	1504	1699	1730	1825	1763	1885	1747	1865	1854	1952	1737	1870	1953	2009
*P. ramiflorus*	1505	1489	1645	1640	1755	1766	1642	1636	2050	2069	1703	1699	2259	2264
*P. rhynchanthus*	388	148	615	326	561	211	622	349	610	314	627	342	626	312
*P. schiedeanus*	1154	1131	1368	1405	1471	1499	1383	1426	1606	1610	1363	1405	1857	1860
*P. sonorae*	253	203	195	141	200	147	184	143	203	143	204	154	205	125

### 3.4 Niche Quantification

**FIGURE 4:**
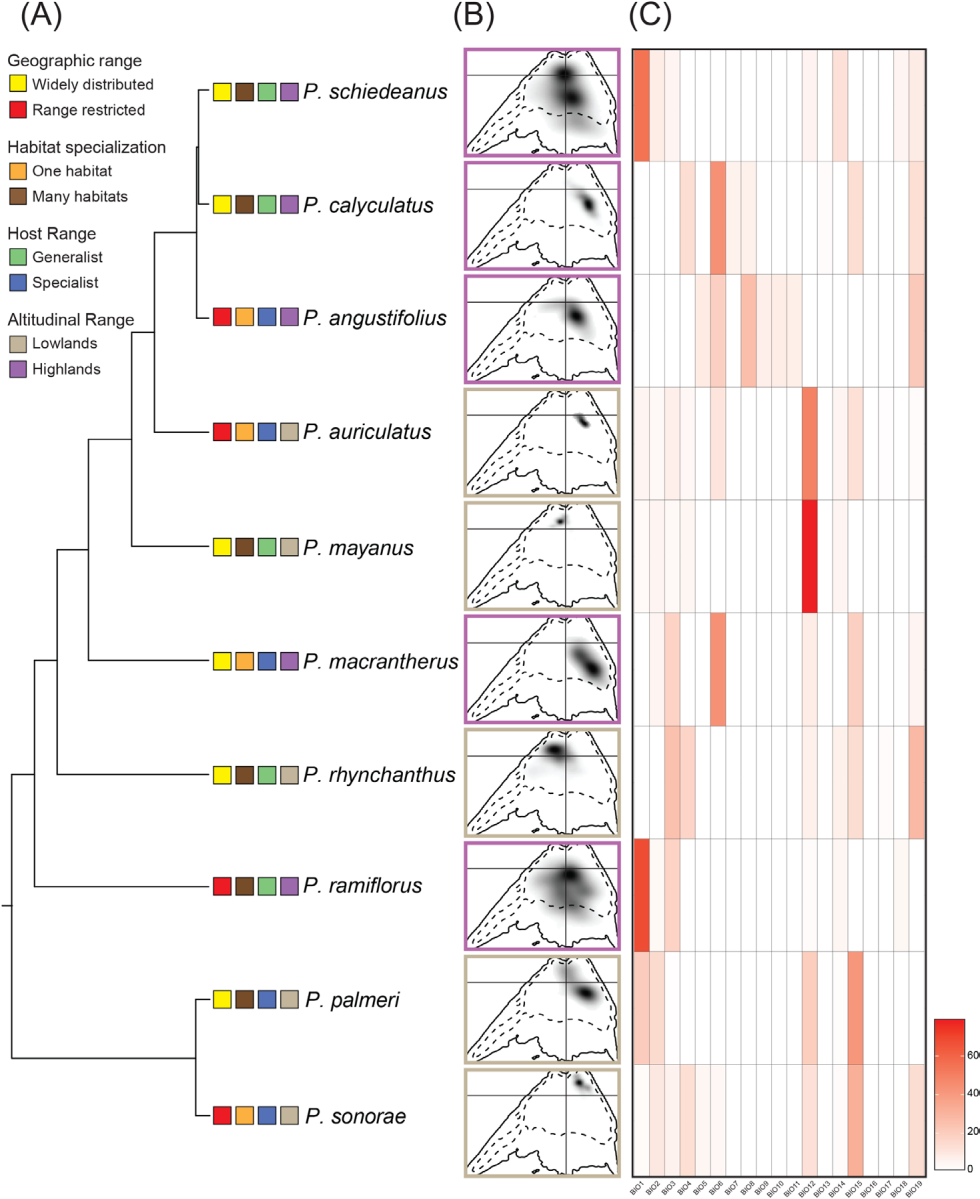
(A) Phylogenetic tree of *Psittacanthus* mistletoes along with (B) results of each mistletoe species niche displayed on the same multi‐dimensional scale represented by the first two axes of a principal components analysis (PCA) summarizing the entire study area. Gray shadings show the density of the occurrences of the species by cell. The solid and dashed contour lines illustrate 100% and 50% of the available (background) environment, respectively. (C) Heatmap showing the BIO variables (BIO1–BIO19) that most contributed for each of the *Psittacanthus* species models using the average values of 10 replicates, where warmer colors indicate higher variable value. Tree phylogeny (A) was derived from a working phylogeny led by Ornelas et al. (2024). *Psittacanthus* mistletoe species coded in the phylogeny by geographic range (widely distributed and range restricted), habitat specialization (one habitat and many habitats), host range (generalist and specialist), and by elevational range (lowlands and highlands). Results presented in the left panel (light brown) correspond to species distributed in xeric and tropical environments at lower elevations and the right panel (purple) to species distributed in more temperate forests at higher elevations. See species coding in Table 1.

Niche density estimations showed that the position in the environmental space varied among species (Figure 4; Table S1). Species from xeric or tropical environments at lower elevations, regardless of geographic range size ( *P. auriculatus* , *P. mayanus* , and *P. sonorae* ), had low niche densities, whereas species distributed in cloud forests, oak forests, pine‐oak forests, or pine‐fir forests at higher elevations ( *P. angustifolius* , *P. macrantherus*, *P*. *ramiflorus*, and *P. schiedeanus*) had high‐density niches (Figure 4). According to niche density surfaces, species with low‐density niches ( *P. auriculatus* , *P*. *calyculatus*, *P. palmeri* , *P. sonorae* ) were those that expanded their distribution area under the different climate change scenarios, whereas species with high‐density niches ( *P. angustifolius* , *P*. *macrantherus*, *P*. *ramiflorus*, *P*. *schiedeanus*) lost suitable area in their distribution area (Figure 2, Figure S1; Table 1). The environmental space of species from temperate forests at higher elevations was mainly related to temperature variables (BIO1, BIO6, BIO8). In contrast, those from more xeric or more tropical environments at lower elevations were mainly related to precipitation variables (BIO12, BIO15, BIO19) (Figure 4).

Niche overlap varied greatly among *Psittacanthus* species, with Schoener's *D* values ranging from 0.000 to 0.677 and Warren's *I* values from 0.000 to 0.827 (Tables S5 and S6). The highest niche overlap occurred between *P*. *ramiflorus* and *P*. *schiedeanus* (*D* = 0.677, *I* = 0.826) and between *P. auriculatus* and *P. palmeri* (*D* = 0.543, *I* = 0.727). In contrast, the lowest niche overlap values were observed between *P. sonorae* and *P. angustifolius* (*D* = 0, *I* = 0) and between *P. sonorae* and P. *mayanus* (*D* = 0, *I* = 0), ecologically contrasting species (Tables S5 and S6).

Niche similarity tests indicate that Schoener's *D* values between *Psittacanthus* species were not significantly higher than those of the null distributions, suggesting that niche overlap between species is explained by regional similarities or differences in available habitat, rather than species interactions or shared evolutionary history (Table S6). In contrast, niches occupied by the 10 *Psittacanthus* species were significantly non‐equivalent (*p* < 0.05), except for the comparisons between *P*. *schiedeanus* and *P*. *ramiflorus* (*p* = 0.089) and *P. angustifolius* and *P*. *rhynchanthus* (*p* < 0.337; Table S6).

## 4 Discussion

Several studies have already established that global climate change will be one of the main causes triggering the redistribution of species, negatively impacting biodiversity and altering ecosystem dynamics (Fontúrbel et al. 2021; Habibullah et al. 2022; Zu et al. 2021). In this study, we focused on both narrowly distributed and geographically widespread *Psittacanthus* species in Mesoamerica. We assessed how climate change under optimistic SSP 2.6 and pessimistic SSP 8.5 scenarios would alter suitable habitat for *Psittacanthus* species by 2050, 2070, and 2090. Specifically, we tested whether species restricted to specialized habitats and narrower geographical distributions and host ranges face greater climate vulnerability than generalist widespread species.

### 4.1 Climate Change Impact on the Distribution of *Psittacanthus* Mistletoes in Mesoamerica

Warming temperatures and decreasing precipitation triggered by climate change (IPCC 2013) elicit four types of species responses: (1) persistence, due to acclimatization and phenotypic plasticity, (2) evolution (local adaptation), (3) migration, or (4) extinction (Aitken et al. 2008; Bussotti et al. 2015). Our results showed that the current distribution of suitable habitat for most species inhabiting temperate forests shifted to higher elevations under most pessimistic scenarios, but their range sizes largely decreased (except *P*. *calyculatus*). Upslope elevation range shifts in *Psittacanthus* species are ubiquitous, with evidence of contemporary species migration towards higher elevations in montane environments (Feeley et al. 2013; Hollenbeck and Sax 2024; Ramírez‐Barahona et al. 2025; Zu et al. 2021). In fact, plants from tropical mountains are expected to migrate upslope in response to warming due to the shallow latitudinal temperature gradient, leading to directional changes in community composition with elevation (e.g., Chen et al. 2011; Colwell et al. 2008; Gottfried et al. 2012; Lenoir et al. 2008; Morueta‐Holme et al. 2015). Recently, Ramírez‐Barahona et al. (2025) analyzed shifts in elevation ranges of plant species in Mesoamerican cloud forests using four decades of species' occurrence records. They found that contemporary upslope shifts are driven by the upslope retreat of the less thermophilic montane species, accompanied by retreating lower and upper edges attributed to varying degrees of species' exposure to deforestation and climate change. The observed elevational shift of the widespread, host generalist *Psittacanthus schiedeanus* inhabiting cloud forests of eastern and southern Mexico supports the findings by Ramírez‐Barahona et al. (2025) for cloud‐forest plant species in Mesoamerica (see also Ornelas, Licona‐Vera, and Ortiz‐Rodriguez 2018). Largely decreased range sizes and elevational shifts were also observed in species inhabiting oak, pine‐oak, pine, and fir forests at higher elevations ( *P. angustifolius* , *P*. *macrantherus*, *P*. *ramiflorus*), suggesting that upslope shifts might also occur in other Mesoamerican mountainous systems. Plausible reasons explaining the observed upward shifts include: (1) rising temperature and total annual precipitation decrease are determinants for upward migration along elevational gradients; (2) the average annual temperature increase and annual precipitation decrease might be relatively stronger at lower elevations than at higher elevations pushing upward shifts of lowland species (i.e., less thermophilic species show more pronounced upslope shifts); (3) certain functional traits (e.g., seed dispersal) affect significantly the migration rates of species; and (4) human disturbances influence species elevational distributions.

Contrary to expectations for plant species from arid zones with low‐density niches (Rajaud and de Noblet‐Decoudré 2017), the suitable habitat areas of *P. auriculatus* , *P. palmeri* , and *P. sonorae* expanded without significant elevational shifts. However, their niches neither overlap nor exhibit similarity or equivalency, suggesting different degrees of environmental heterogeneity among arid zones. The fact that annual precipitation (BIO12) and precipitation seasonality (BIO15) substantially contributed to the models of these *Psittacanthus* species could be related to the humidity received from the sea and/or microhabitats found in the surrounding canyons and sierras, ranging from some of the driest to somewhat wetter conditions (see also Miguel‐Vázquez et al. 2023). Although future warming can severely affect both hosts and mistletoes (Bowers and Turner 2001; Fontúrbel 2020; Spurrier and Smith 2007), humidity could help the hosts and the *Psittacanthus* species by lessening the extreme conditions in arid regions, such as high temperatures or droughts, and the water demands of their interaction.

The decreased range sizes of habitat suitable in tropical lowlands for *P. mayanus* and *P*. *rhynchanthus* were minimal. It is possible that the marked area contraction of suitable habitat and elevational shift in *P. mayanus* resulted from the collapse of the summer precipitation regime, longstanding droughts, and aridification of the Yucatán Peninsula, a biogeographic region historically characterized by these climatic oscillations (Licona‐Vera et al. 2018). For *P*. *rhynchanthus*, phenotypic plasticity might explain the absence of or limited range shift or expansion in the wet tropical lowlands. However, evidence for latitudinal or longitudinal range shifts in the lowland tropics where species are likely to be closer to their rainfall tolerance limits is almost inexistent (reviewed in Lenoir and Svenning 2015). Global change research should become a priority for plants in tropical lowland ecosystems, where long‐term data are often scarce, to investigate the apparent lack of lowland range shifts and constraints limiting climate‐change‐driven lowland range shifts (Lenoir and Svenning 2015).

### 4.2 Testing the Vulnerability of *Psittacanthus* by Host and Habitat Specialization

Specialist species might be disproportionately vulnerable to habitat loss and climate change due to the synergistic effects of a narrow niche and small range size (Brown 1984; Slatyer et al. 2013). In this sense, we expected that range‐restricted mistletoe species with narrow environmental tolerances would be most threatened by climate change as compared to widely distributed species. However, our models projected a potential increase in suitable habitat for most of the range‐restricted species examined. Using the mistletoe threat index (Fadini et al. 2024) and range size, we hypothesized that the following traits predispose species to greater climate change threat: endemism, occurrence in a single habitat type, a narrow host range, restricted distribution, and reliance on a single pollinator or seed disperser. Consequently, we predict that such specialist mistletoes will experience greater range declines than generalists. This hypothesis was not fully supported in our study. For instance, endemic range‐restricted *P. auriculatus* to one xeric habitat and parasitizing mainly one host plant species (Díaz Infante et al. 2016; Ornelas, Licona‐Vera, and Vásquez‐Aguilar 2018), gained over 200% of its current distribution area, the greatest expansion in range area of all studied *Psittacanthus* species. Despite its generalist ecology, evidenced by a broad host range, wide distribution, and presence across multiple habitat types (Licona‐Vera et al. 2018), *P. mayanus* suffered the most severe range contraction, losing over 97% of its suitable area. In contrast, *P. angustifolius* , a specialist mistletoe occurring only in pine forests that are capable of parasitizing few host plant species (*Pinus* spp.), and with a restricted distribution in northern Mesoamerica (Howell and Mathiasen 2004; Mathiasen et al. 2000; Mathiasen et al. 2007), lost over 76% of its current distribution area, supporting our hypothesis based on Fadini et al.'s (2024) mistletoe threat index. Similarly, *P*. *calyculatus*, a generalist with a much wider host range and widely distributed geographically and across different habitat types (Arce‐Acosta et al. 2016; Díaz Infante et al. 2016; Lara et al. 2021; Pérez‐Crespo et al. 2017; Rodríguez‐Mendieta et al. 2018), gained distribution area to over 70% of its current distribution. Given that the vulnerability of the studied *Psittacanthus* species was not fully explained by their host and habitat specialization, the vulnerability, or not, of the studied *Psittacanthus* species might be linked to land‐use changes, habitat vulnerability, and/or vulnerability and hydric stress of host species (Fontúrbel 2020).

### 4.3 Other Factors Contributing to Range and Elevational Shifts

Range‐restricted, host‐specialist *Psittacanthus* species were expected to decline under future warming scenarios. However, *P*. *calyculatus* showed a northward range shift and moderate elevational changes under both optimistic and pessimistic projections. This suggests a positive response to global warming because (1) its exceptionally broad host range, with more than 50 tree species of diverse genera reported to be parasitized (Arce‐Acosta et al. 2016; Díaz Infante et al. 2016; Kuijt 2009; Pérez‐Crespo, Lara, and Ornelas 2016); (2) its current widespread geographic distribution and apparent tolerance to disturbance linked to distribution in edges of temperate forests and human‐modified landscapes (Arce‐Acosta et al. 2016; Díaz Infante et al. 2016; Pérez‐Crespo, Ornelas, et al. 2016; Zuria et al. 2014); (3) seed dispersal by both resident and migratory bird species (Díaz Infante et al. 2016; Lara et al. 2009, 2021; Rodríguez‐Mendieta et al. 2018; Zuria et al. 2014); and (4) anthropogenic land‐use changes whose rates are particularly high along the Trans‐Mexican Volcanic Belt (Aguilar‐Tomasini et al. 2020; Bravo‐Espinosa et al. 2014; Khan et al. 2021). An alternative scenario is that *P*. *calyculatus* migrates towards cooler places by switching host species (Tikkanen et al. 2021; Watson et al. 2022; Zamora and Mellado 2019). However, this is a challenging task for mistletoes, as they depend on other species, which may have different dispersal abilities to shift their distribution ranges (Rai et al. 2018; Sangüesa‐Barreda et al. 2018) and, thus, the persistence of mistletoes would be precluded by spatial mismatches with their hosts, pollinators, and seed dispersers (Fontúrbel 2020; Ornelas, Licona‐Vera, and Ortiz‐Rodriguez 2018).

The range area of species inhabiting xeric environments ( *P. auriculatus* , *P. palmeri* , *P. sonorae* ) expanded in the future, with little changes in elevation (see also Ornelas, Licona‐Vera, and Vásquez‐Aguilar 2018; Ornelas, Licona‐Vera, and Ortiz‐Rodriguez 2018). This result contradicts studies that predict range size reductions and no migration to higher elevations for plant species inhabiting xeric environments (Aguirre et al. 2017; Duarte et al. 2019; Gomes Vale and Brito 2015; Rajaud and de Noblet‐Decoudré 2017). The observation of range expansion might be simply the result of increased dryland expansion rate, as observed in other regions, leading to reduced carbon sequestration and enhanced warming over the present drylands (Duarte et al. 2019; Huang et al. 2015; Rajaud and de Noblet‐Decoudré 2017). Other factors contributing to increased range size areas include: (1) potential shifts to less frequently parasitized host species where both mistletoe and host species overlap their suitable areas (Lira‐Noriega and Peterson 2014), from *Acacia schaffneri* to *Prosopis laevigata* and *Acacia pennatula* for *P. auriculatus* (Díaz Infante et al. 2016; Ornelas, Licona‐Vera, and Vásquez‐Aguilar 2018; Peguero and Espelta 2011), from *Bursera microphylla* to less frequently parasitized *Bursera* species and *Cyrtocarpa edulis* along the arid coastal strip and sand dunes for *P. sonorae* (Ornelas, Licona‐Vera, and Ortiz‐Rodriguez 2018; Ornelas et al. 2019; Overton 1994), and from *Bursera* species to *Erythrina* species for *P. palmeri* (Kuijt 2009); (2) tracking phenological changes of Phainopeplas ( *Phainopepla nitens* ), a seasonal migrant between the Sonoran Desert and semiarid woodlands in surrounding regions south to central Mexico, and seed disperser of all three desert‐adapted *Psittacanthus* species during winter (Díaz Infante et al. 2016; Overton 1994; Sandoval‐Ortega et al. 2022); and (3) mistletoe traits such as the seasonal deciduousness of *P. palmeri* , small size and succulent fleshy stems of *P. sonorae* , and seed latency in *P. auriculatus* and *P. sonorae* , in which cotyledons remain unexpanded encapsulated by dried viscin and seed coat to inhabit in extremely arid environments, are clearly adaptations to dry ecosystems (Ornelas et al. 2024; Ortiz‐Rodriguez et al. 2018).

## 5 Conclusions

Understanding climate vulnerability for keystone resources, such as mistletoes, is an important first step in developing conservation proposals. Our study provides novel and significant insights into the impact of climate change on Mesoamerican *Psittacanthus* species distribution and the role of their climatic niches. We showed that *Psittacanthus* species will respond differently depending on the environment in which they are distributed. The divergent responses predicted for *Psittacanthus* species to climate change are consistent with environmental characterization and results of niche similarity tests, which highlight altogether the environmental uniqueness that each species occupies. In this context, the *Psittacanthus* species' environmental distinctiveness turns into an exciting sentinel model to explore the responses that climatically contrasting plant communities would have to climate change.

## Author Contributions

**Antonio Acini Vásquez‐Aguilar:** conceptualization (equal), data curation (equal), formal analysis (equal), investigation (equal), methodology (equal), validation (equal), visualization (equal), writing – original draft (equal), writing – review and editing (equal). **Saddan Morales‐Saldaña:** conceptualization (equal), data curation (equal), formal analysis (equal), investigation (equal), methodology (equal), supervision (equal), validation (equal), visualization (equal), writing – original draft (equal), writing – review and editing (equal). **Samantha Maite de los Santos‐Gómez:** data curation (equal), formal analysis (equal), investigation (equal), methodology (equal), visualization (equal), writing – review and editing (equal). **Andrea I. Barraza‐Ochoa:** formal analysis (equal), methodology (equal), visualization (equal), writing – review and editing (equal). **Juan Francisco Ornelas:** conceptualization (equal), funding acquisition (equal), investigation (equal), methodology (equal), project administration (equal), resources (equal), supervision (equal), validation (equal), visualization (equal), writing – original draft (equal), writing – review and editing (equal).

## Conflicts of Interest

The authors declare no conflicts of interest.

## Supporting information


**Table S1:** Characteristics and occurrence data of *Psittacanthus* species included in this study.
**Table S2:** Bioclimatic variables used in MaxEnt for the modeling of current and future distribution of habitat suitability of *Psittacanthus* mistletoe species.
**Table S3:** WorldClim variables used for the construction of the distribution and ecological niche modeling for *Psittacanthus* species.
**Table S4:** Calibration results of different clusters based on kuenm_ceval function of the kuenm package in R.
**Table S5:** Niche overlap values between *Psittacanthus* mistletoe species using Schoener's *D* (below diagonal) and Warren's *I* indexes (above diagonal). Niche overlap is represented from 0 (no overlap) to 1 (full overlap).
**Table S6:** Ecological niche comparisons for *Psittacanthus* species.
**Figure S1:** Predicted distribution of *Psittacanthus* mistletoe species under climate‐change scenarios, from optimistic SSP 2.6 Shared Socioeconomic Pathways conditions for 2050, 2070, and 2090. (A) *Psittacanthus angustifolius*, (B) *P. auriculatus* , (C) *P*. *calyculatus*, (D) *P*. *macrantherus*, (E) *P. mayanus* . Predicted distribution of *Psittacanthus* mistletoe species under climate‐change scenarios, from optimistic SSP 2.6 Shared Socioeconomic Pathways conditions for 2050, 2070, and 2090. (F) *Psittacanthus palmeri*, (G) *P*. *ramiflorus*, (H) *P*. *rhynchanthus*, (I) *P*. *schiedeanus*, (J) *P. sonorae* .

## Data Availability

Occurrence data points (geographic coordinates and elevation), shape files (.shp, M masks), R scripts, and the readme file are available on the Zenodo repository (https://doi.org/10.5281/zenodo.15307295).
