# Supplementary material for: Range and Elevational Shifts of Mistletoes Under Future Climate Change Scenarios
Source: Ecol Evol. 2025 Oct 28;15(10):e72388. doi: 10.1002/ece3.72388 (PMC12559679; doi:10.1002/ece3.72388)

This material serves as supplementary content for the research article titled:

"Range and Elevational Shifts of Mistletoes Under Future Climate Change Scenarios"

by

Antonio Acini Vásquez-Aguilar, Saddan Morales-Saldaña, Samantha Maite de los Santos-Gómez, Andrea I. Barraza-Ochoa, and Juan Francisco Ornelas (DOI: 10.0000/xxx.00000).

Supporting Information

Characteristics and occurrence data of *Psittacanthus* species included in this study (**Table S1**), bioclimatic variables used in MaxEnt for modelling of current and future distribution of habitat suitability (**Table S2**), WorldClim variables used for construction of the distribution and ecological niche modelling (**Table S3**), calibration results of different clusters based on kuenm_ceval function (**Table S4**), niche overlap values using Schoener's *D* and Warren's *I* indexes (**Table S5**), ecological niche comparisons for *Psittacanthus* species (**Table S6**), and predicted distribution of *Psittacanthus* mistletoe species under climate-change scenarios, from optimistic SSP 2.6 conditions for 2050, 2070 and 2090 (**Figure S1**).

TABLES

TABLE S1 | Characteristics and occurrence data of *Psittacanthus* species included in this study.

Species	Geographical range	Habitat specialization	Host range	Elevational range	Presence records
Psittacanthus angustifolius Kuijt	R	O	S	H	52
Psittacanthus auriculatus Eichler	R	O	S	L	43
Psittacanthus calyculatus G.Don	W	M	G	H	716
Psittacanthus macrantherus Eichler	W	O	S	H	52
Psittacanthus mayanus Standl. & Steyerm.	W	M	G	L	118
Psittacanthus palmeri (Watson) Barlow & Wiens	W	M	S	L	85
Psittacanthus ramiflorus G.Don	R	M	G	H	162
Psittacanthus rhynchanthus (Benth.) Kuijt	W	M	G	L	242
Psittacanthus schiedeanus (Cham. & Schltdl.) G.Don	W	M	G	H	200
Psittacanthus sonorae (S.Watson) Kuijt	R	O	S	L	181

W = widely distributed, R = range restricted, O = one habitat type, M = many habitat types, S = host specialist, G = host generalist, L = lowlands (< 1000 m above sea level), H = highlands (> 1000 m above sea level).

TABLE S2 | Bioclimatic variables used in MaxEnt for the modeling of current and future distribution of habitat suitability of *Psittacanthus* mistletoe species.

Code	Description	Unit
BIO1	Annual Mean Temperature	°C
BIO2	Mean Diurnal Range (Mean of monthly (max temp-min temp)	°C
BIO3	Isothermality ((BIO2/BIO7)*100)	%
BIO4	Temperature Seasonality (standard deviation*100)	%
BIO5	Maximum Temperature of Warmest Month	°C
BIO6	Min Temperature of Coldest Month	°C
BIO7	Temperature Annual Range (BIO5-BIO6)	°C
BIO8	Mean Temperature of Wettest Quarter	°C
BIO9	Mean Temperature of Driest Quarter	°C
BIO10	Mean Temperature of Warmest Quarter	°C
BIO11	Mean Temperature of Coldest Quarter	°C
BIO12	Annual Precipitation	mm
BIO13	Precipitation of Wettest Month	mm
BIO14	Precipitation of Driest Month (Coefficient of Variation)	mm
BIO15	Precipitation Seasonality	%
BIO16	Precipitation of Wettest Quarter	mm
BIO17	Precipitation of Driest Quarter	mm
BIO18	Precipitation of Warmest Quarter	mm
BIO19	Precipitation of Coldest Quarter	mm

TABLE S3 | WorldClim variables used for the construction of the distribution and ecological niche modelling for *Psittacanthus* species.

**Species**	**WorldClim variables**
P. angustifolius	BIO1, BIO5, BIO6, BIO8, BIO9, BIO10, BIO11, BIO19
P. auriculatus	BIO1, BIO2, BIO3, BIO4, BIO6, BIO12, BIO14, BIO15, BIO17, BIO19
P. calyculatus	BIO4, BIO6, BIO7, BIO8, BIO13, BIO15, BIO19
P. macrantherus	BIO1, BIO5, BIO6, BIO8, BIO9, BIO10, BIO11, BIO14, BIO15, BIO17
P. mayanus	BIO1, BIO2, BIO3, BIO4, BIO5, BIO12, BIO14, BIO18
P. palmeri	BIO1, BIO2, BIO3, BIO12, BIO14, BIO15, BIO18, BIO19
P. ramiflorus	BIO1, BIO2, BIO3, BIO12, BIO14, BIO18
P. rhynchanthus	BIO3, BIO4, BIO12, BIO14, BIO15, BIO17, BIO19
P. schiedeanus	BIO1, BIO2, BIO3, BIO12, BIO14, BIO18, BIO19
P. sonorae	BIO1, BIO2, BIO3, BIO4, BIO5, BIO6, BIO8, BIO12, BIO14, BIO15, BIO19

TABLE S4 | Calibration results of different clusters based on kuenm_ceval function of the kuenm package in R.

**Species**	**Feature Class**	**Regularization multiplier**	**Species**	**Partial ROC**	**Omission rate 5%**	**AICc**	**Delta AICc**
P. angustifolius	L, Q	0.5	1.73	0	0	1236.967	0
P. auriculatus	L	0.1	1.890	0	0	866.876	0.000
P. calyculatus	L, Q, P	0.1	1.44	0	0.034	17613.23	0
P. macrantherus	L, T	1.5	1.56	0	0	1239.867	0
P. mayanus	L, Q	1.7	1.437	0	0.029	2857.724	0
P. palmeri	Q, L, T	1.3	1.276	0	0	1509.811	0.000
P. ramiflorus	L, Q, P	1.5	1.612	0	0.042	3864.509	0.000
P. rhynchanthus	Q, P	0.3	1.31	0	0.04	6743.8	0
P. schiedeanus	L, Q, P	0.3	1.389	0	0.05	5062.527	0
P. sonorae	L, Q, P, T	1.7	1.903	0	0.143	3636.524	0

TABLE S5 | Niche overlap values between *Psittacanthus* mistletoe species using Schoener's *D* (below diagonal) and Warren's *I* indexes (above diagonal). Niche overlap is represented from 0 (no overlap) to 1 (full overlap). Niche overlap between species > 0.2 in yellow.

Species	*angustifolius*	*auriculatus*	*calyculatus*	*macrantherus*	*mayanus*	*palmeri*	*ramiflorus*	*rhynchanthus*	*schiedeanus*	*sonorae*
angustifolius		0.1136713	0.1410302	0.2892140	0.0709755	0.1748781	0.2240857	0.3593105	0.1947544	0.0000026
auriculatus	0.0469648		0.4741464	0.2652384	0.0302567	0.5183022	0.0735025	0.0746600	0.0507266	0.0196866
calyculatus	0.0806521	0.2408979		0.5567950	0.0380387	0.7278722	0.1080789	0.0693727	0.0633375	0.1079461
macrantherus	0.1781996	0.0789082	0.3305927		0.0179458	0.3560019	0.1213912	0.0679577	0.0826929	0.0315042
mayanus	0.0291541	0.0231735	0.0125067	0.0062942		0.2007066	0.0342129	0.2860833	0.0490416	0.0000002
palmeri	0.1180290	0.2872070	0.5437027	0.2058246	0.0099648		0.0944282	0.1572940	0.0814355	0.1688221
ramiflorus	0.0547321	0.0064225	0.0230532	0.0354486	0.0028229	0.0178403		0.1718441	0.8269902	0.0052207
rhynchanthus	0.1917479	0.0162614	0.0376157	0.0407353	0.1044487	0.0882178	0.0818532		0.1481933	0.0026097
schiedeanus	0.0396319	0.0033604	0.0126029	0.0216514	0.0034115	0.0126615	0.6765964	0.0567479		0.0063052
sonorae	0.0000000	0.0052821	0.0589639	0.0138693	0.0000000	0.1049803	0.0010919	0.0017116	0.0008524	

Table S6 | Ecological niche comparisons for *Psittacanthus* species. Niche overlap between species > 0.2 in yellow.

Comparisons		Niche overlap (*D*)	Niche similarity		Niche equivalency
a	b		a-b	b-a	
P. angustifolius	*P. auriculatus*	0.047	0.911 ns	0.901 ns	0.009 **
P. angustifolius	*P. calyculatus*	0.080	0.931 ns	0.891 ns	0.009 **
P. angustifolius	*P. macrantherus*	0.178	0.980 ns	0.911 ns	0.009 **
P. angustifolius	*P. mayanus*	0.029	0772 ns	0.683 ns	0.009 **
P. angustifolius	*P. palmeri*	0.118	0.931 ns	0.950 ns	0.009 **
P. angustifolius	*P. ramiflorus*	0.054	0.950 ns	0.980 ns	0.009 **
P. angustifolius	*P. rhynchanthus*	0.192	0.842 ns	0.832 ns	0.337 ns
P. angustifolius	*P. schiedeanus*	0.039	1.000 ns	0.980 ns	0.009 **
P. angustifolius	*P. sonorae*	0.000	0.149 ns	0.208 ns	0.009 **
P. auriculatus	*P. calyculatus*	0.241	0.921 ns	0.950 ns	0.009 **
P. auriculatus	*P. macrantherus*	0.078	0.931 ns	0.931 ns	0.009 **
P. auriculatus	*P. mayanus*	0.023	0.782 ns	0.772 ns	0.009 **
P. auriculatus	*P. palmeri*	0.287	1.000 ns	0.990 ns	0.009 **
P. auriculatus	*P. ramiflorus*	0.006	0.941 ns	0.960 ns	0.009 **
P. auriculatus	*P. rhynchanthus*	0.016	0.782 ns	0.733 ns	0.009 **
P. auriculatus	*P. schiedeanus*	0.003	0.881 ns	0.891 ns	0.009 **
P. auriculatus	*P. sonorae*	0.005	0.710 ns	0.733 ns	0.009 **
P. calyculatus	*P. macrantherus*	0.331	0.980 ns	0.950 ns	0.009 **
P. calyculatus	*P. mayanus*	0.012	0.663 ns	0.703 ns	0.009 **
P. calyculatus	*P. palmeri*	0.544	0.980 ns	0.950 ns	0.009 **
P. calyculatus	*P. ramiflorus*	0.023	0.931 ns	0.901 ns	0.009 **
P. calyculatus	*P. rhynchanthus*	0.037	0.653 ns	0.713 ns	0.009 **
P. calyculatus	*P. schiedeanus*	0.012	0.871ns	0.802 ns	0.009 **
P. calyculatus	*P. sonorae*	0.059	0.792 ns	0.772 ns	0.009 **
P. macrantherus	*P. mayanus*	0.006	0.663 ns	0.584 ns	0.009 **
P. macrantherus	*P. palmeri*	0.206	0.941 ns	0.960 ns	0.009 **
P. macrantherus	*P. ramiflorus*	0.035	0.901 ns	0.931 ns	0.009 **
P. macrantherus	*P. rhynchanthus*	0.040	0.723 ns	0.683 ns	0.009 **
P. macrantherus	*P. schiedeanus*	0.217	0.822 ns	0.842 ns	0.009 **
P. macrantherus	*P. sonorae*	0.013	0.703 ns	0.683 ns	0.009 **
P. mayanus	*P. palmeri*	0.078	0.990 ns	1.000 ns	0.009 **
P. mayanus	*P. ramiflorus*	0.002	0.891 ns	0.921 ns	0.009 **
P. mayanus	*P. rhynchanthus*	0.104	0.871 ns	0.941 ns	0.009 **
P. mayanus	*P. schiedeanus*	0.003	0.941 ns	0.891 ns	0.009 **
P. mayanus	*P. sonorae*	0.000	0.347 ns	1.000 ns	0.009 **
P. palmeri	*P. ramiflorus*	0.017	0.871 ns	0.921 ns	0.009 **
P. palmeri	*P. rhynchanthus*	0.088	0.792 ns	0.772 ns	0.009 **
P. palmeri	*P. schiedeanus*	0.012	0.842 ns	0.881 ns	0.009 **
P. palmeri	*P. sonorae*	0.105	0.752 ns	0.772 ns	0.009 **
P. ramiflorus	*P. rhynchanthus*	0.081	0.584 ns	0.782 ns	0.009 **
P. ramiflorus	*P. schiedeanus*	0.677	1.000 ns	0.960 ns	0.089 ns
P. ramiflorus	*P. sonorae*	0.001	0.149 ns	0.2 83 ns	0.009 **
P. rhynchanthus	*P. schiedeanus*	0.056	0.802 ns	0.832 ns	0.009 **
P. rhynchanthus	*P. sonorae*	0.001	0.277 ns	0.248 ns	0.009 **
P. schiedeanus	*P. sonorae*	0.000	0.267 ns	0.158 ns	0.009 **

FIGURES

FIGURE S1 | Predicted distribution of *Psittacanthus* mistletoe species under climate-change scenarios, from optimistic SSP 2.6 Shared Socioeconomic Pathways conditions for 2050, 2070 and 2090. (A) *Psittacanthus angustifolius*, (B) *P. auriculatus*, (C) *P. calyculatus*, (D) *P. macrantherus*, (E) *P. mayanus*.

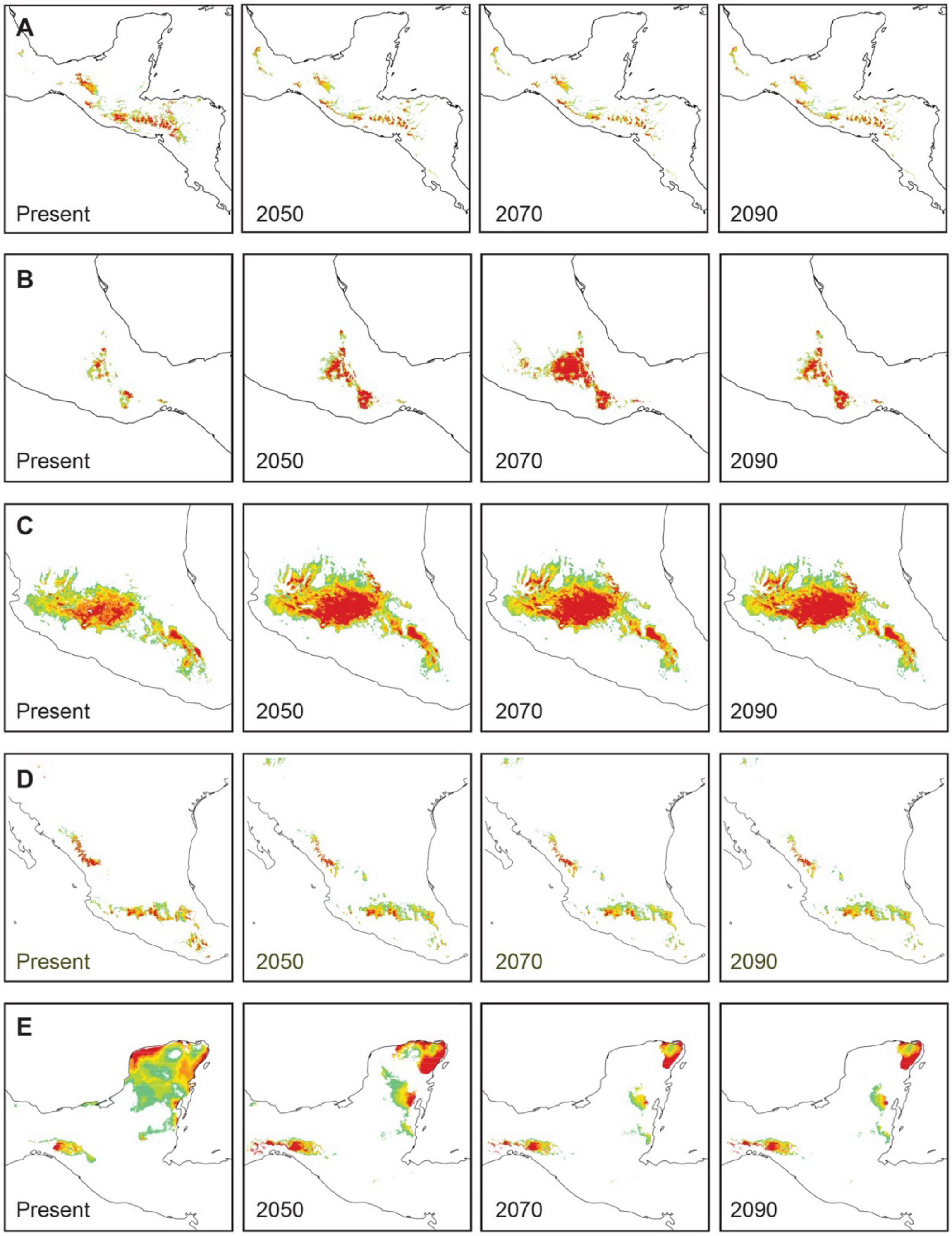

FIGURE S1 | Continued…Predicted distribution of *Psittacanthus* mistletoe species under climate-change scenarios, from optimistic SSP 2.6 Shared Socioeconomic Pathways conditions for 2050, 2070 and 2090. (F) *Psittacanthus palmeri*, (G) *P. ramiflorus*, (H) *P. rhynchanthus*, (I) *P. schiedeanus*, (J) *P. sonorae*.

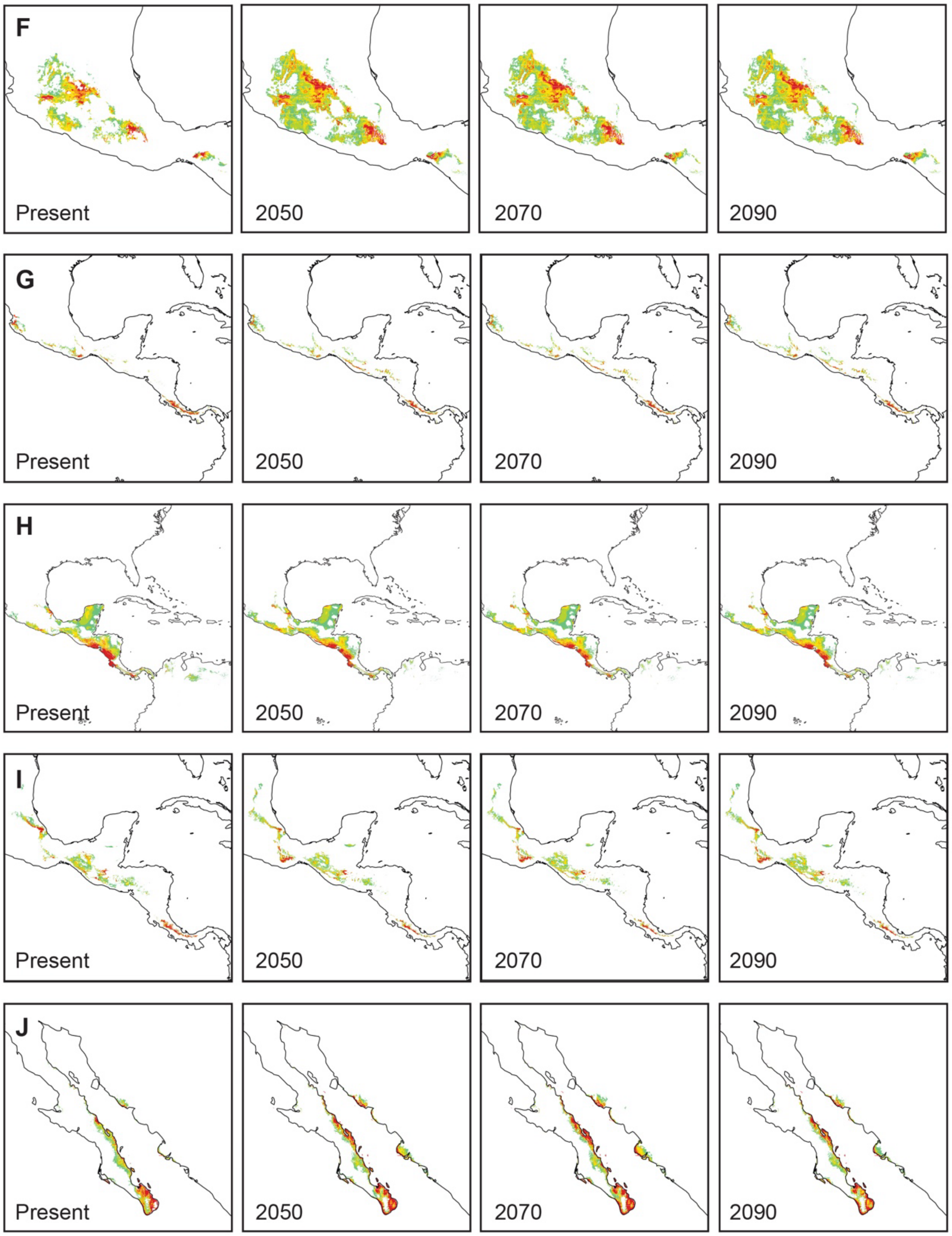

Supplement: Supplementary file 1 — Table S1: Characteristics and occurrence data of Psittacanthus species included in this study. Table S2: Bioclimatic variables used in MaxEnt for the modeling of current and future distribution of habitat suitability of Psittacanthus mistletoe species. Table S3: WorldClim variables used for the construction of the distribution and ecological niche modeling for Psittacanthus species. Table S4: Calibration results of different clusters based on kuenm_ceval function of the kuenm package in R. Table S5: Niche overlap values between Psittacanthus mistletoe species using Schoener's D (below diagonal) and Warren's I indexes (above diagonal). Niche overlap is represented from 0 (no overlap) to 1 (full overlap). Table S6: Ecological niche comparisons for Psittacanthus species. Figure S1: Predicted distribution of Psittacanthus mistletoe species under climate‐change scenarios, from optimistic SSP 2.6 Shared Socioeconomic Pathways conditions for 2050, 2070, and 2090. (A) Psittacanthus angustifolius, (B) P. auriculatus , (C) P. calyculatus, (D) P. macrantherus, (E) P. mayanus . Predicted distribution of Psittacanthus mistletoe species under climate‐change scenarios, from optimistic SSP 2.6 Shared Socioeconomic Pathways conditions for 2050, 2070, and 2090. (F) Psittacanthus palmeri, (G) P. ramiflorus, (H) P. rhynchanthus, (I) P. schiedeanus, (J) P. sonorae . [file ECE3-15-e72388-s001.pdf]
